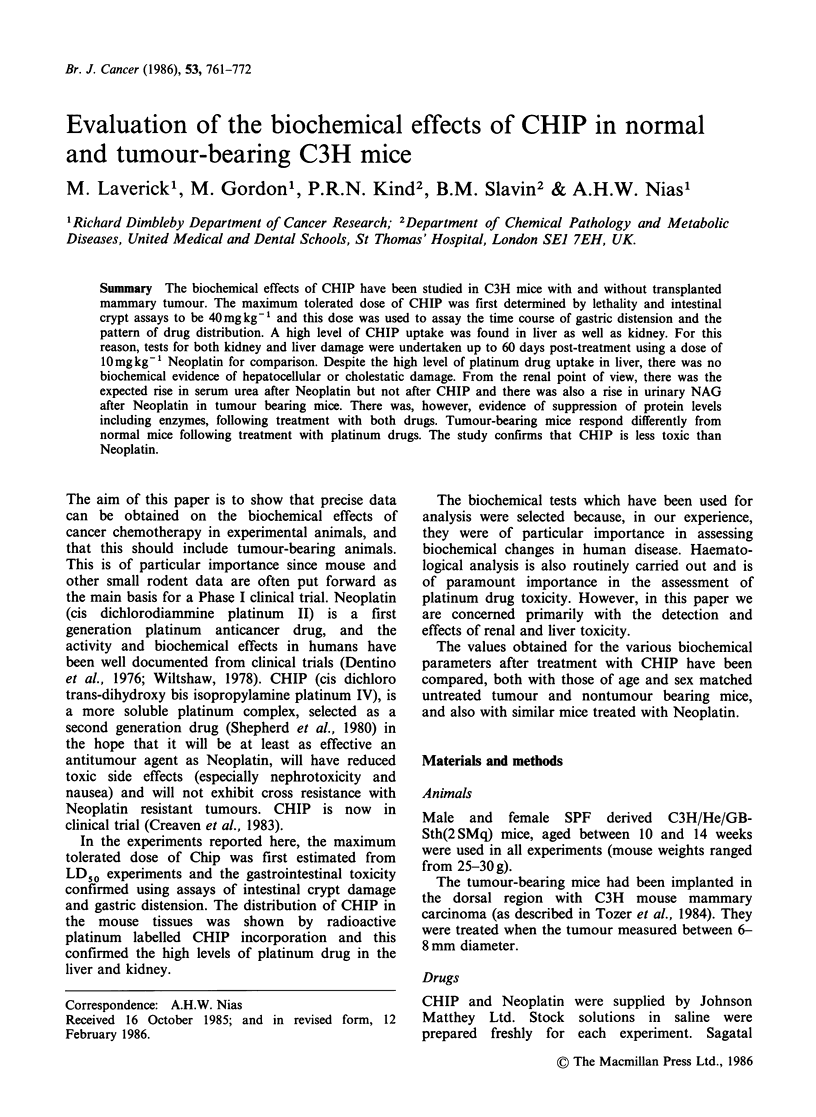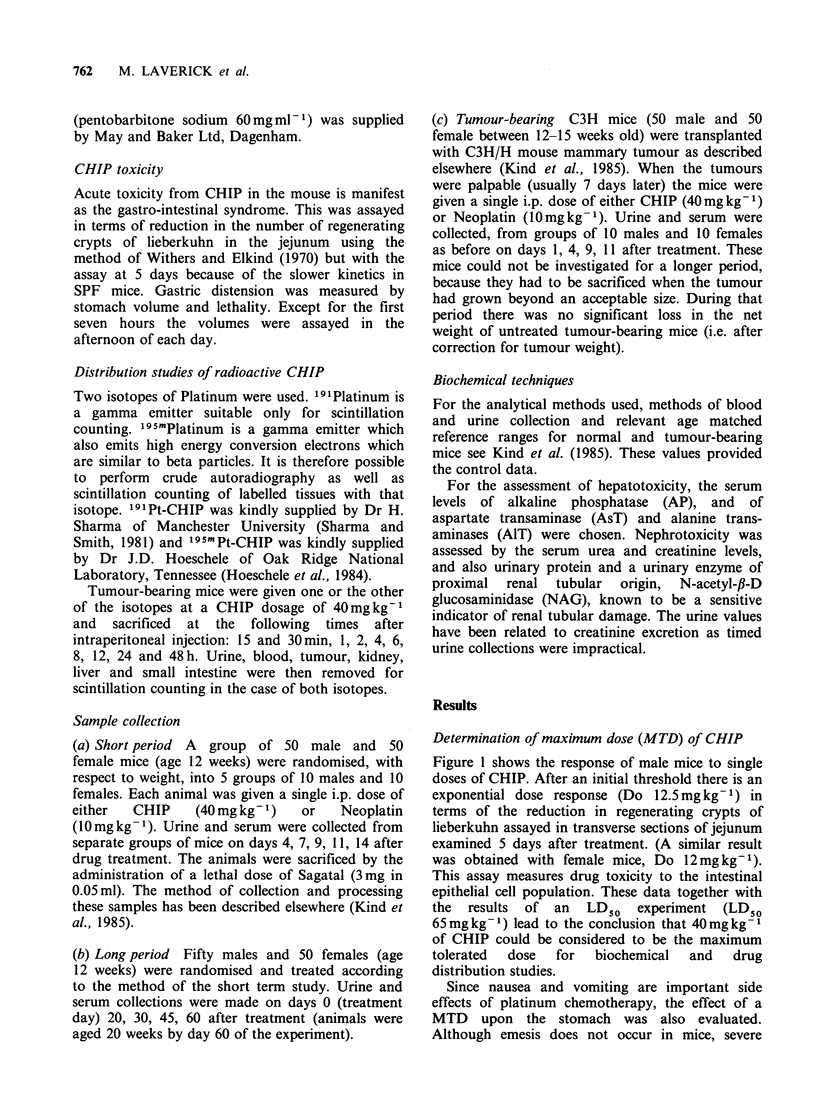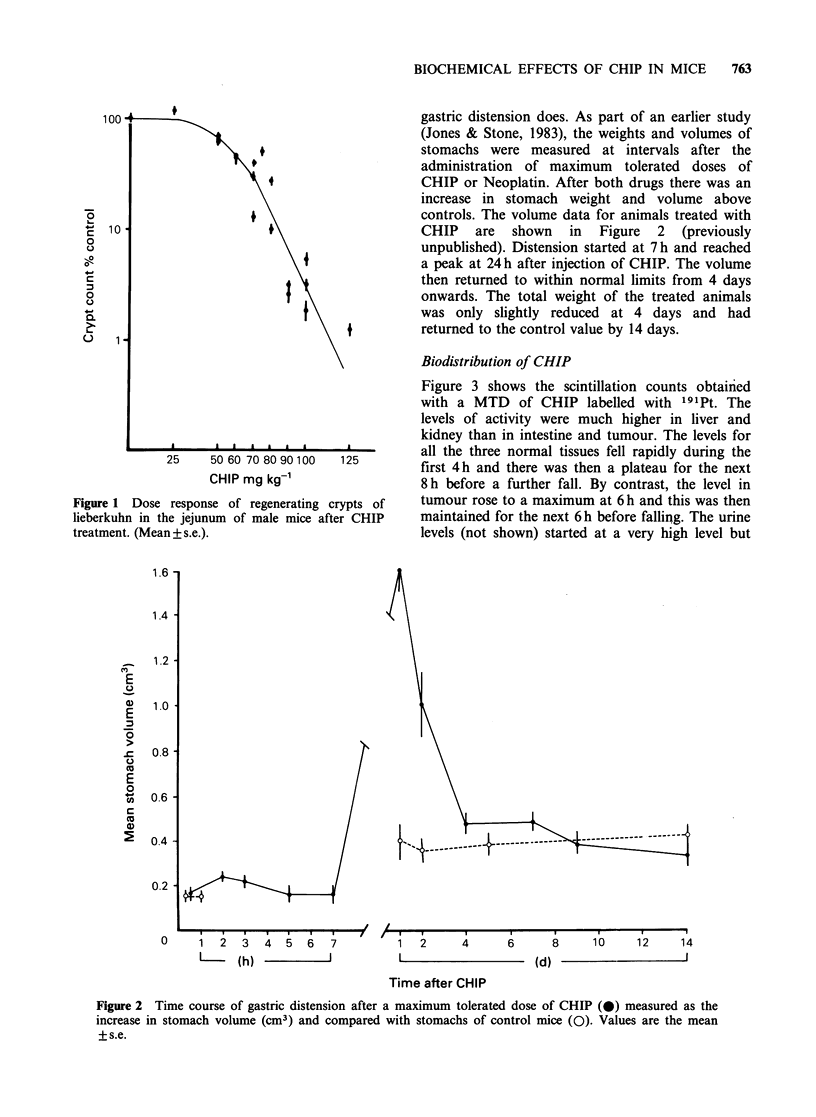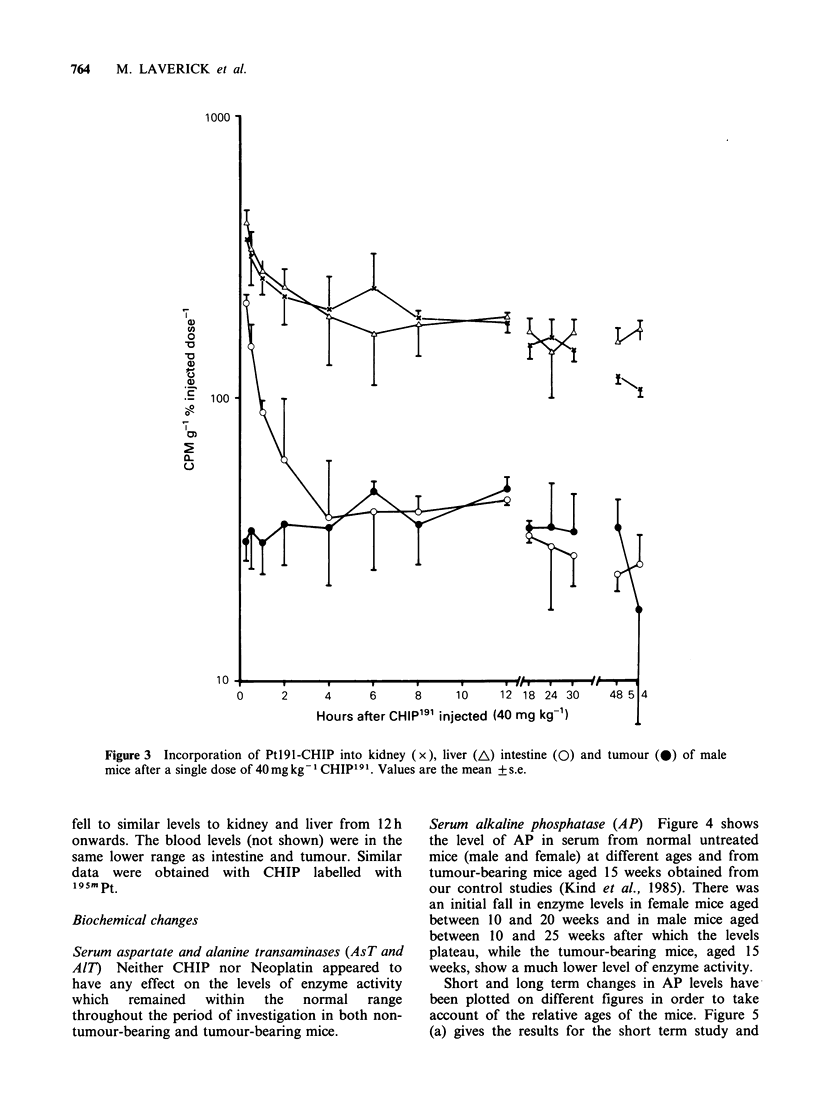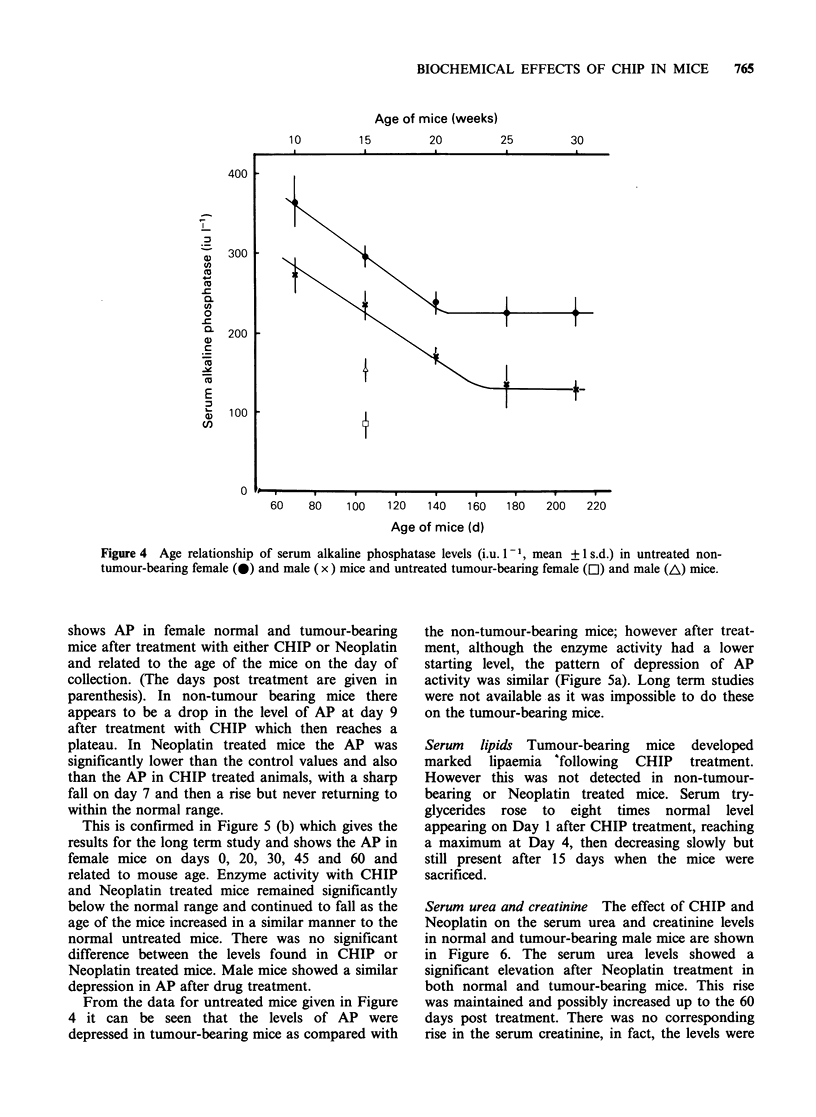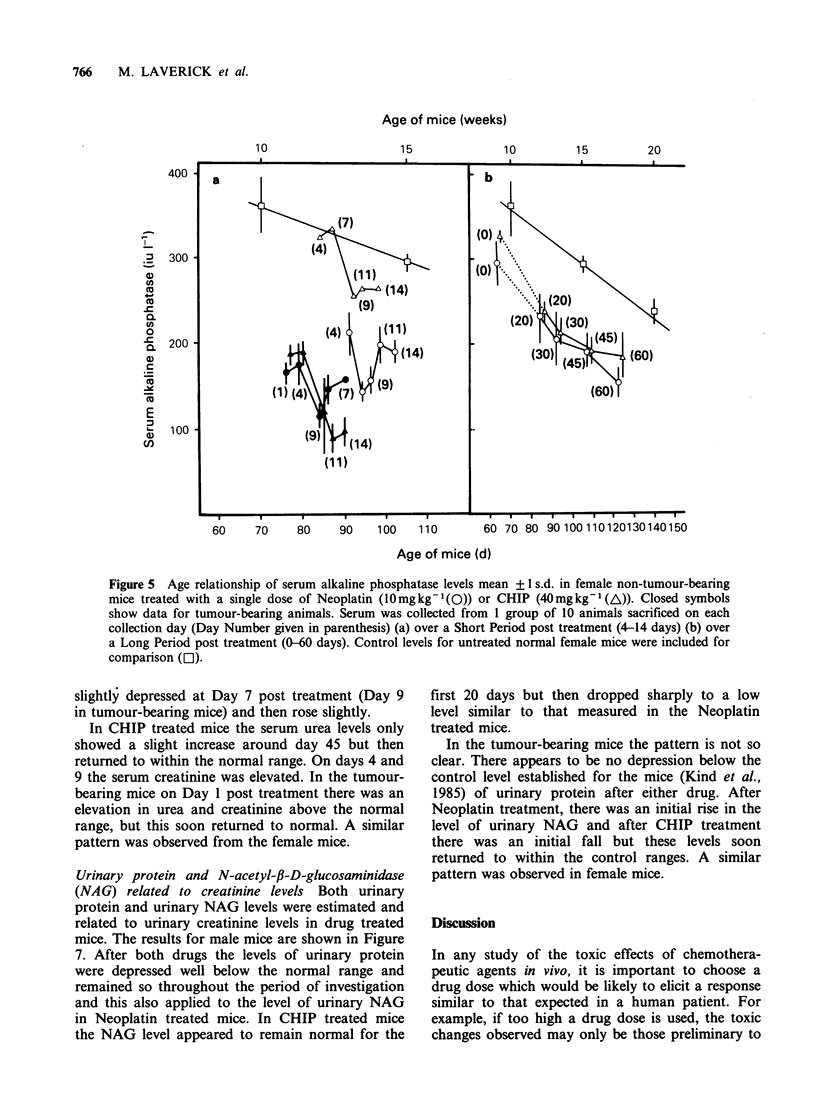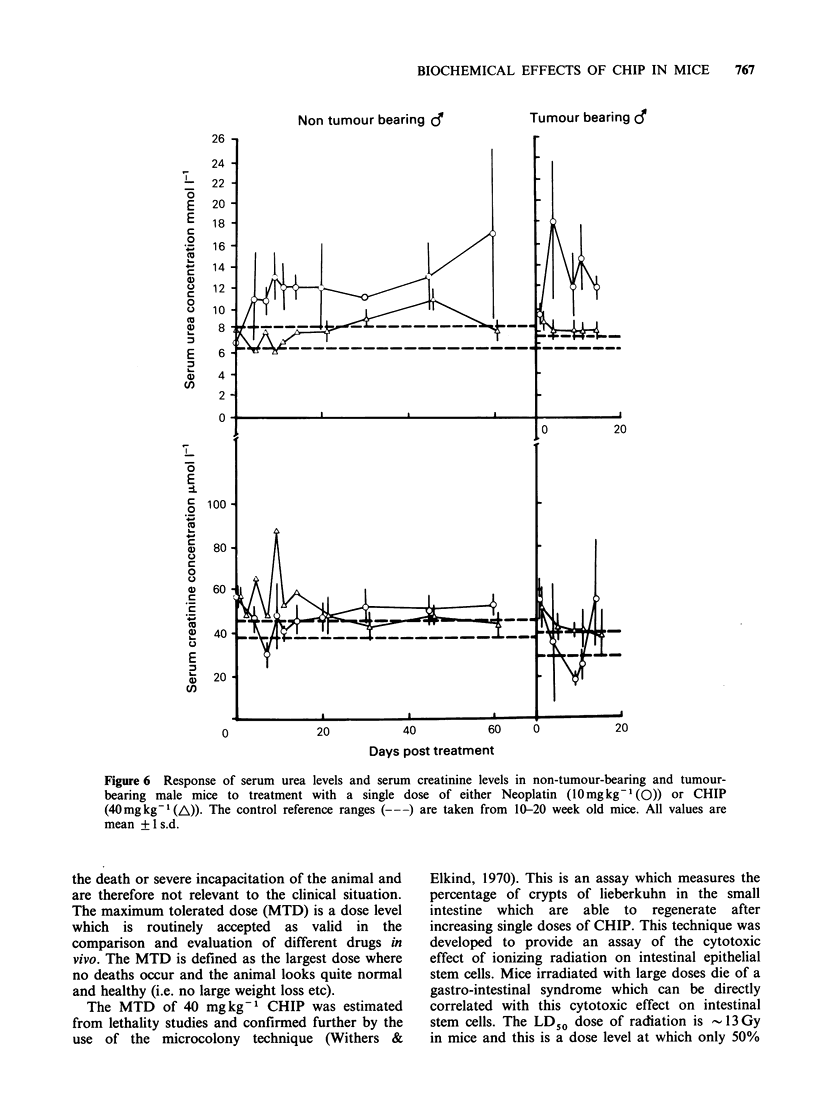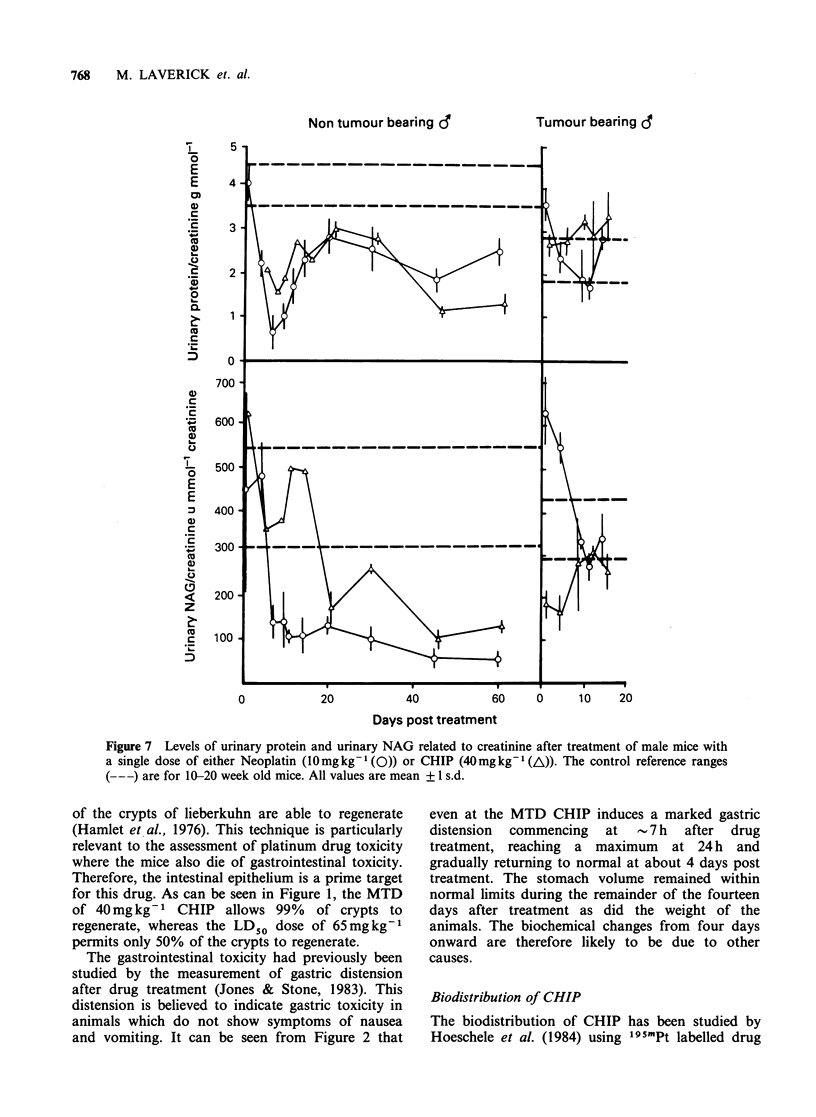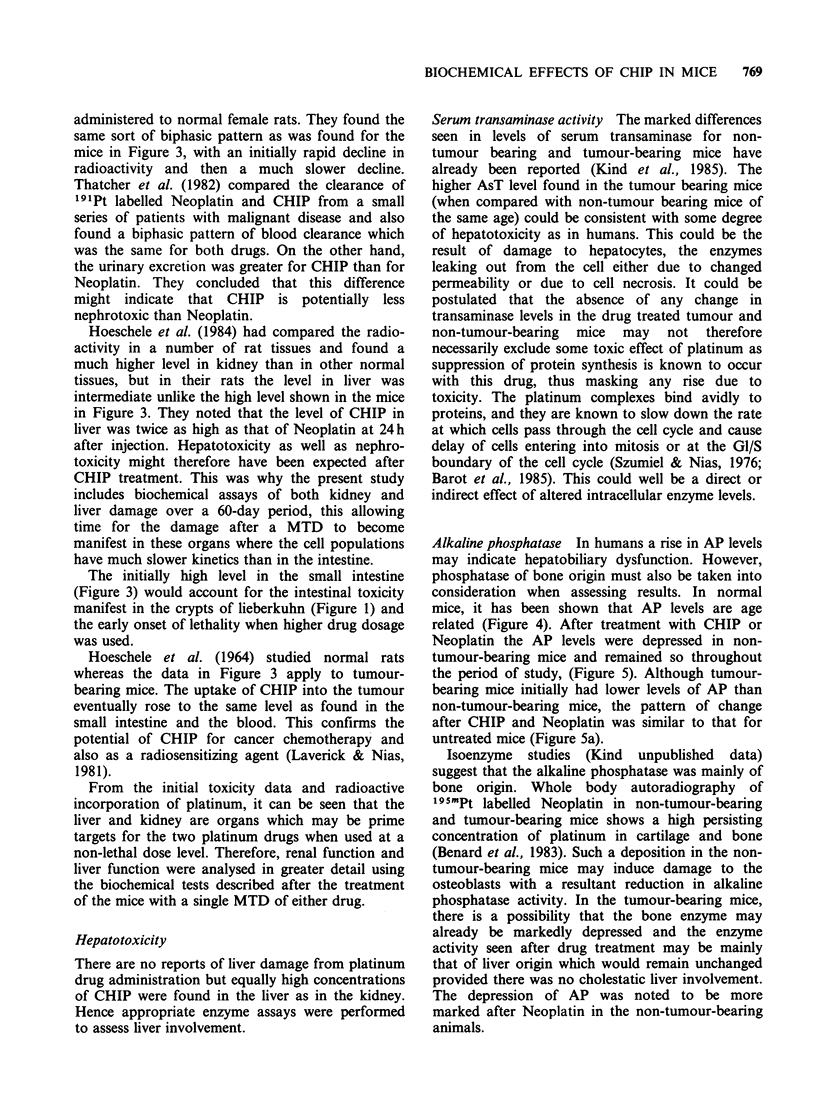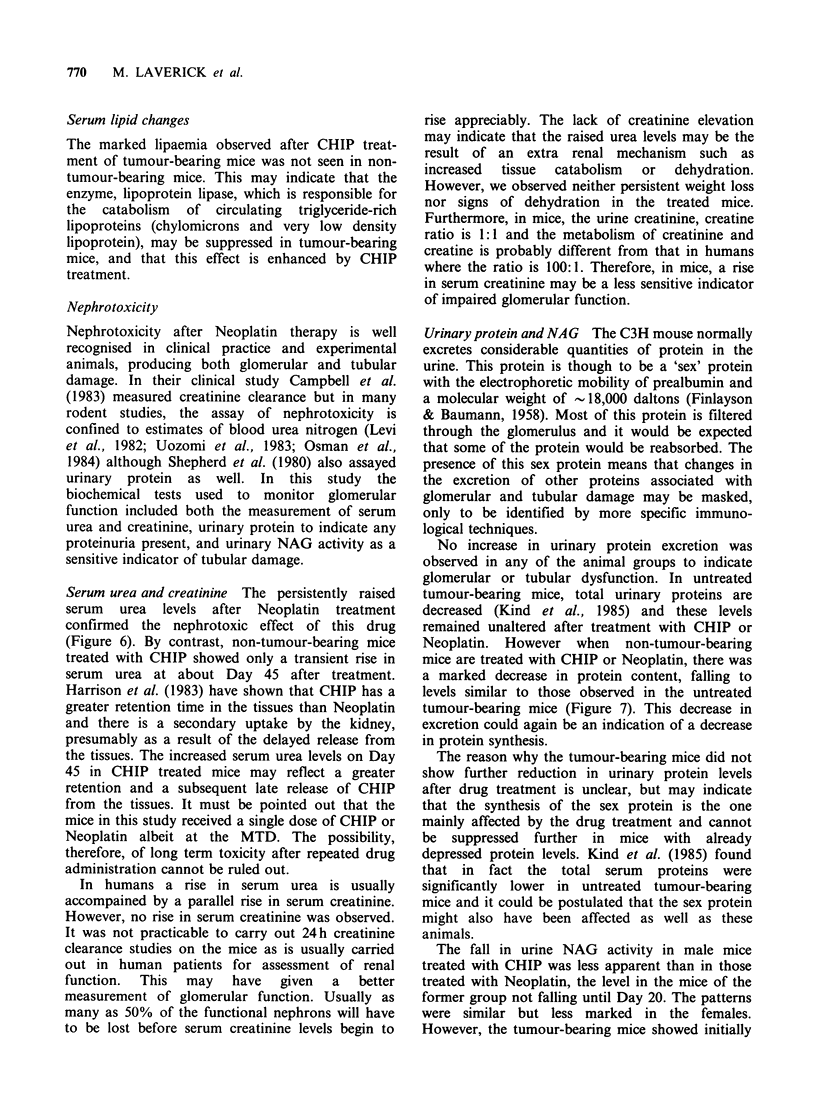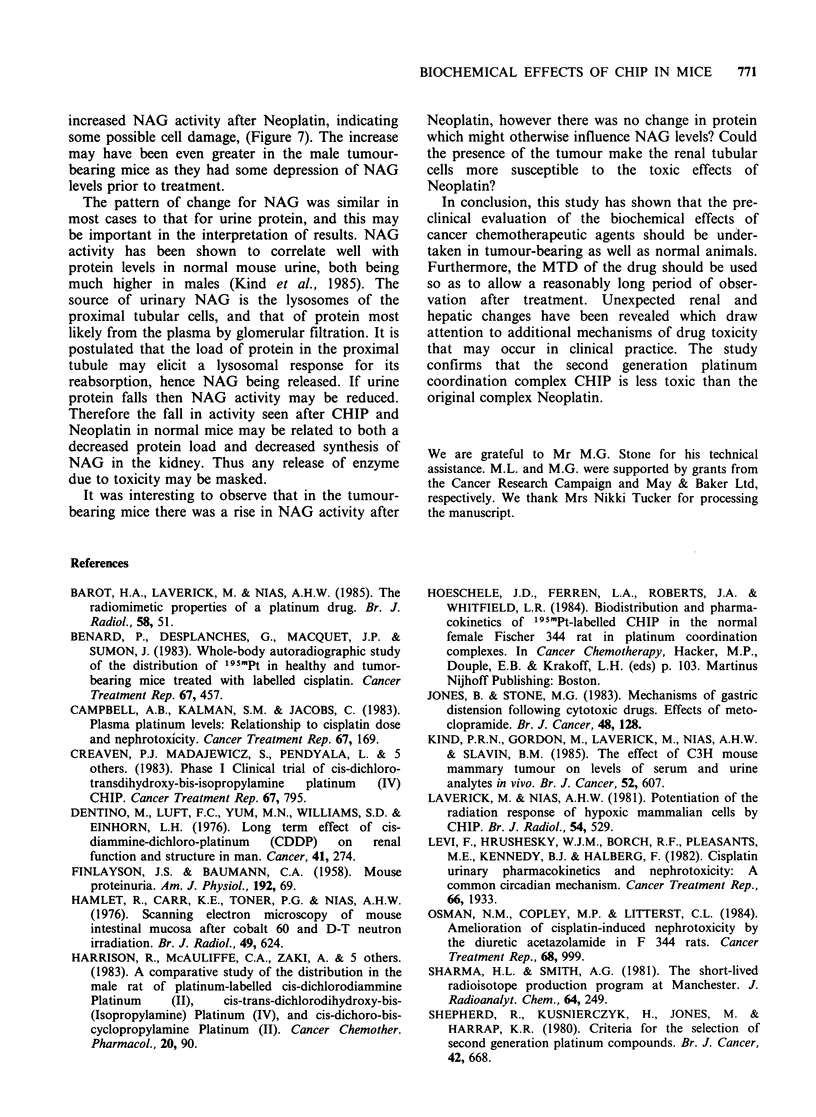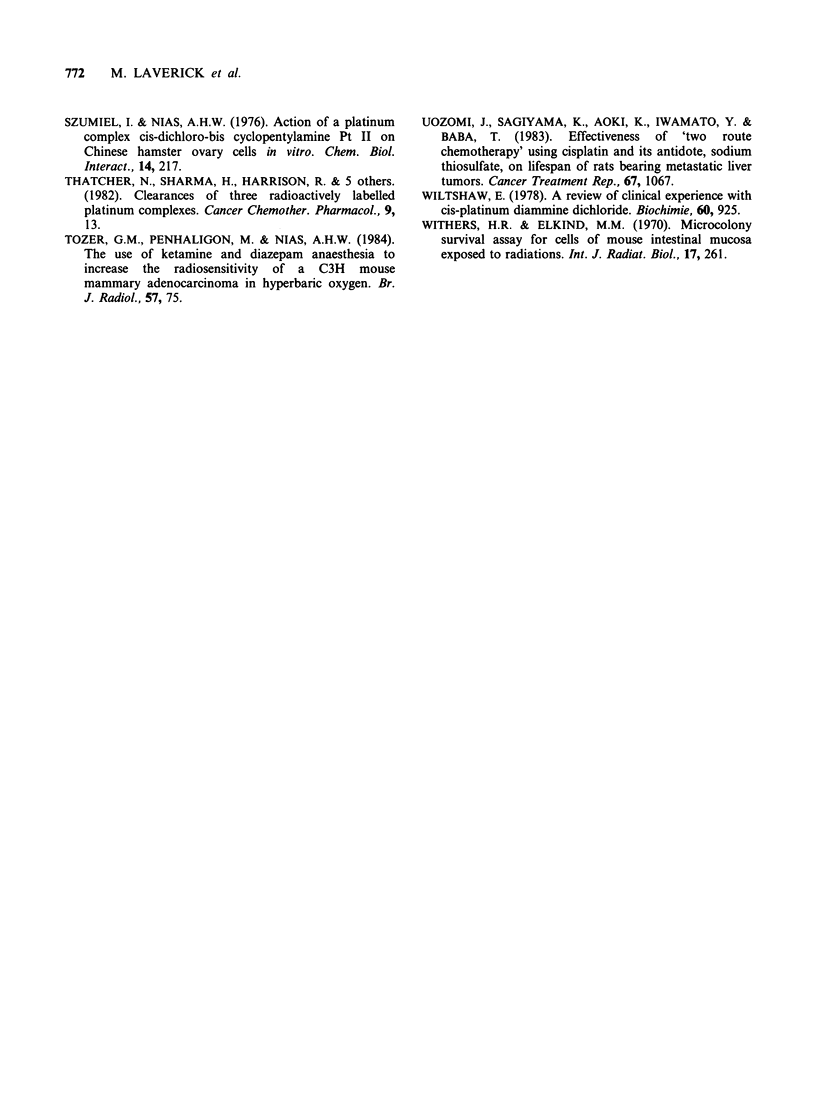# Evaluation of the biochemical effects of CHIP in normal and tumour-bearing C3H mice.

**DOI:** 10.1038/bjc.1986.130

**Published:** 1986-06

**Authors:** M. Laverick, M. Gordon, P. R. Kind, B. M. Slavin, A. H. Nias

## Abstract

The biochemical effects of CHIP have been studied in C3H mice with and without transplanted mammary tumour. The maximum tolerated dose of CHIP was first determined by lethality and intestinal crypt assays to be 40 mg kg-1 and this dose was used to assay the time course of gastric distension and the pattern of drug distribution. A high level of CHIP uptake was found in liver as well as kidney. For this reason, tests for both kidney and liver damage were undertaken up to 60 days post-treatment using a dose of 10 mg kg-1 Neoplatin for comparison. Despite the high level of platinum drug uptake in liver, there was no biochemical evidence of hepatocellular or cholestatic damage. From the renal point of view, there was the expected rise in serum urea after Neoplatin but not after CHIP and there was also a rise in urinary NAG after Neoplatin in tumour bearing mice. There was, however, evidence of suppression of protein levels including enzymes, following treatment with both drugs. Tumour-bearing mice respond differently from normal mice following treatment with platinum drugs. The study confirms that CHIP is less toxic than Neoplatin.


Br. J. Cancer (1986), 53, 761–772

### Evaluation of the biochemical effects of CHIP in normal and tumour-bearing C3H mice

M. Laverick[1], M. Gordon[1], P.R.N. Kind[2], B.M. Slavin[2] & A.H.W. Nias[1]

[1]*Richard Dimbleby Department of Cancer Research;* [2]*Department of Chemical Pathology and Metabolic Diseases, United Medical and Dental Schools, St Thomas' Hospital, London SE1 7EH, UK.*

**Summary** The biochemical effects of CHIP have been studied in C3H mice with and without transplanted mammary tumour. The maximum tolerated dose of CHIP was first determined by lethality and intestinal crypt assays to be $40\,mg\,kg^{-1}$ and this dose was used to assay the time course of gastric distension and the pattern of drug distribution. A high level of CHIP uptake was found in liver as well as kidney. For this reason, tests for both kidney and liver damage were undertaken up to 60 days post-treatment using a dose of $10\,mg\,kg^{-1}$ Neoplatin for comparison. Despite the high level of platinum drug uptake in liver, there was no biochemical evidence of hepatocellular or cholestatic damage. From the renal point of view, there was the expected rise in serum urea after Neoplatin but not after CHIP and there was also a rise in urinary NAG after Neoplatin in tumour bearing mice. There was, however, evidence of suppression of protein levels including enzymes, following treatment with both drugs. Tumour-bearing mice respond differently from normal mice following treatment with platinum drugs. The study confirms that CHIP is less toxic than Neoplatin.

The aim of this paper is to show that precise data can be obtained on the biochemical effects of cancer chemotherapy in experimental animals, and that this should include tumour-bearing animals. This is of particular importance since mouse and other small rodent data are often put forward as the main basis for a Phase I clinical trial. Neoplatin (cis dichlorodiammine platinum II) is a first generation platinum anticancer drug, and the activity and biochemical effects in humans have been well documented from clinical trials (Dentino *et al.*, 1976; Wiltshaw, 1978). CHIP (cis dichloro trans-dihydroxy bis isopropylamine platinum IV), is a more soluble platinum complex, selected as a second generation drug (Shepherd *et al.*, 1980) in the hope that it will be at least as effective an antitumour agent as Neoplatin, will have reduced toxic side effects (especially nephrotoxicity and nausea) and will not exhibit cross resistance with Neoplatin resistant tumours. CHIP is now in clinical trial (Creaven *et al.*, 1983).

In the experiments reported here, the maximum tolerated dose of Chip was first estimated from $LD_{50}$ experiments and the gastrointestinal toxicity confirmed using assays of intestinal crypt damage and gastric distension. The distribution of CHIP in the mouse tissues was shown by radioactive platinum labelled CHIP incorporation and this confirmed the high levels of platinum drug in the liver and kidney.

Correspondence: A.H.W. Nias
Received 16 October 1985; and in revised form, 12 February 1986.

The biochemical tests which have been used for analysis were selected because, in our experience, they were of particular importance in assessing biochemical changes in human disease. Haematological analysis is also routinely carried out and is of paramount importance in the assessment of platinum drug toxicity. However, in this paper we are concerned primarily with the detection and effects of renal and liver toxicity.

The values obtained for the various biochemical parameters after treatment with CHIP have been compared, both with those of age and sex matched untreated tumour and nontumour bearing mice, and also with similar mice treated with Neoplatin.

### Materials and methods

### *Animals*

Male and female SPF derived C3H/He/GB-Sth(2SMq) mice, aged between 10 and 14 weeks were used in all experiments (mouse weights ranged from 25–30 g).

The tumour-bearing mice had been implanted in the dorsal region with C3H mouse mammary carcinoma (as described in Tozer *et al.*, 1984). They were treated when the tumour measured between 6–8 mm diameter.

### *Drugs*

CHIP and Neoplatin were supplied by Johnson Matthey Ltd. Stock solutions in saline were prepared freshly for each experiment. Sagatal

© The Macmillan Press Ltd., 1986

(pentobarbitone sodium 60 mg ml$^{-1}$) was supplied by May and Baker Ltd, Dagenham.

*CHIP toxicity*

Acute toxicity from CHIP in the mouse is manifest as the gastro-intestinal syndrome. This was assayed in terms of reduction in the number of regenerating crypts of lieberkuhn in the jejunum using the method of Withers and Elkind (1970) but with the assay at 5 days because of the slower kinetics in SPF mice. Gastric distension was measured by stomach volume and lethality. Except for the first seven hours the volumes were assayed in the afternoon of each day.

*Distribution studies of radioactive CHIP*

Two isotopes of Platinum were used. $^{191}$Platinum is a gamma emitter suitable only for scintillation counting. $^{195m}$Platinum is a gamma emitter which also emits high energy conversion electrons which are similar to beta particles. It is therefore possible to perform crude autoradiography as well as scintillation counting of labelled tissues with that isotope. $^{191}$Pt-CHIP was kindly supplied by Dr H. Sharma of Manchester University (Sharma and Smith, 1981) and $^{195m}$Pt-CHIP was kindly supplied by Dr J.D. Hoeschele of Oak Ridge National Laboratory, Tennessee (Hoeschele et al., 1984).

Tumour-bearing mice were given one or the other of the isotopes at a CHIP dosage of 40 mg kg$^{-1}$ and sacrificed at the following times after intraperitoneal injection: 15 and 30 min, 1, 2, 4, 6, 8, 12, 24 and 48 h. Urine, blood, tumour, kidney, liver and small intestine were then removed for scintillation counting in the case of both isotopes.

*Sample collection*

(*a*) *Short period* A group of 50 male and 50 female mice (age 12 weeks) were randomised, with respect to weight, into 5 groups of 10 males and 10 females. Each animal was given a single i.p. dose of either CHIP (40 mg kg$^{-1}$) or Neoplatin (10 mg kg$^{-1}$). Urine and serum were collected from separate groups of mice on days 4, 7, 9, 11, 14 after drug treatment. The animals were sacrificed by the administration of a lethal dose of Sagatal (3 mg in 0.05 ml). The method of collection and processing these samples has been described elsewhere (Kind et al., 1985).

(*b*) *Long period* Fifty males and 50 females (age 12 weeks) were randomised and treated according to the method of the short term study. Urine and serum collections were made on days 0 (treatment day) 20, 30, 45, 60 after treatment (animals were aged 20 weeks by day 60 of the experiment).

(*c*) *Tumour-bearing* C3H mice (50 male and 50 female between 12–15 weeks old) were transplanted with C3H/H mouse mammary tumour as described elsewhere (Kind et al., 1985). When the tumours were palpable (usually 7 days later) the mice were given a single i.p. dose of either CHIP (40 mg kg$^{-1}$) or Neoplatin (10 mg kg$^{-1}$). Urine and serum were collected, from groups of 10 males and 10 females as before on days 1, 4, 9, 11 after treatment. These mice could not be investigated for a longer period, because they had to be sacrificed when the tumour had grown beyond an acceptable size. During that period there was no significant loss in the net weight of untreated tumour-bearing mice (i.e. after correction for tumour weight).

*Biochemical techniques*

For the analytical methods used, methods of blood and urine collection and relevant age matched reference ranges for normal and tumour-bearing mice see Kind et al. (1985). These values provided the control data.

For the assessment of hepatotoxicity, the serum levels of alkaline phosphatase (AP), and of aspartate transaminase (AsT) and alanine transaminases (AlT) were chosen. Nephrotoxicity was assessed by the serum urea and creatinine levels, and also urinary protein and a urinary enzyme of proximal renal tubular origin, N-acetyl-$\beta$-D glucosaminidase (NAG), known to be a sensitive indicator of renal tubular damage. The urine values have been related to creatinine excretion as timed urine collections were impractical.

### Results

*Determination of maximum dose (MTD) of CHIP*

Figure 1 shows the response of male mice to single doses of CHIP. After an initial threshold there is an exponential dose response (Do 12.5 mg kg$^{-1}$) in terms of the reduction in regenerating crypts of lieberkuhn assayed in transverse sections of jejunum examined 5 days after treatment. (A similar result was obtained with female mice, Do 12 mg kg$^{-1}$). This assay measures drug toxicity to the intestinal epithelial cell population. These data together with the results of an $LD_{50}$ experiment ($LD_{50}$ 65 mg kg$^{-1}$) lead to the conclusion that 40 mg kg$^{-1}$ of CHIP could be considered to be the maximum tolerated dose for biochemical and drug distribution studies.

Since nausea and vomiting are important side effects of platinum chemotherapy, the effect of a MTD upon the stomach was also evaluated. Although emesis does not occur in mice, severe

**Figure 1** Dose response of regenerating crypts of lieberkuhn in the jejunum of male mice after CHIP treatment. (Mean ± s.e.).

gastric distension does. As part of an earlier study (Jones & Stone, 1983), the weights and volumes of stomachs were measured at intervals after the administration of maximum tolerated doses of CHIP or Neoplatin. After both drugs there was an increase in stomach weight and volume above controls. The volume data for animals treated with CHIP are shown in Figure 2 (previously unpublished). Distension started at 7 h and reached a peak at 24 h after injection of CHIP. The volume then returned to within normal limits from 4 days onwards. The total weight of the treated animals was only slightly reduced at 4 days and had returned to the control value by 14 days.

*Biodistribution of CHIP*

Figure 3 shows the scintillation counts obtained with a MTD of CHIP labelled with $^{191}$Pt. The levels of activity were much higher in liver and kidney than in intestine and tumour. The levels for all the three normal tissues fell rapidly during the first 4 h and there was then a plateau for the next 8 h before a further fall. By contrast, the level in tumour rose to a maximum at 6 h and this was then maintained for the next 6 h before falling. The urine levels (not shown) started at a very high level but

**Figure 2** Time course of gastric distension after a maximum tolerated dose of CHIP (●) measured as the increase in stomach volume (cm$^3$) and compared with stomachs of control mice (○). Values are the mean ± s.e.

**Figure 3** Incorporation of Pt191-CHIP into kidney (×), liver (△) intestine (○) and tumour (●) of male mice after a single dose of $40 \, mg \, kg^{-1}$ $CHIP^{191}$. Values are the mean ±s.e.

fell to similar levels to kidney and liver from 12h onwards. The blood levels (not shown) were in the same lower range as intestine and tumour. Similar data were obtained with CHIP labelled with $^{195m}Pt$.

*Biochemical changes*

*Serum aspartate and alanine transaminases (AsT and AlT)* Neither CHIP nor Neoplatin appeared to have any effect on the levels of enzyme activity which remained within the normal range throughout the period of investigation in both non-tumour-bearing and tumour-bearing mice.

*Serum alkaline phosphatase (AP)* Figure 4 shows the level of AP in serum from normal untreated mice (male and female) at different ages and from tumour-bearing mice aged 15 weeks obtained from our control studies (Kind et al., 1985). There was an initial fall in enzyme levels in female mice aged between 10 and 20 weeks and in male mice aged between 10 and 25 weeks after which the levels plateau, while the tumour-bearing mice, aged 15 weeks, show a much lower level of enzyme activity.

Short and long term changes in AP levels have been plotted on different figures in order to take account of the relative ages of the mice. Figure 5 (a) gives the results for the short term study and

**Figure 4** Age relationship of serum alkaline phosphatase levels (i.u. $l^{-1}$, mean $\pm 1$ s.d.) in untreated non-tumour-bearing female (●) and male (×) mice and untreated tumour-bearing female (□) and male (△) mice.

shows AP in female normal and tumour-bearing mice after treatment with either CHIP or Neoplatin and related to the age of the mice on the day of collection. (The days post treatment are given in parenthesis). In non-tumour bearing mice there appears to be a drop in the level of AP at day 9 after treatment with CHIP which then reaches a plateau. In Neoplatin treated mice the AP was significantly lower than the control values and also than the AP in CHIP treated animals, with a sharp fall on day 7 and then a rise but never returning to within the normal range.

This is confirmed in Figure 5 (b) which gives the results for the long term study and shows the AP in female mice on days 0, 20, 30, 45 and 60 and related to mouse age. Enzyme activity with CHIP and Neoplatin treated mice remained significantly below the normal range and continued to fall as the age of the mice increased in a similar manner to the normal untreated mice. There was no significant difference between the levels found in CHIP or Neoplatin treated mice. Male mice showed a similar depression in AP after drug treatment.

From the data for untreated mice given in Figure 4 it can be seen that the levels of AP were depressed in tumour-bearing mice as compared with the non-tumour-bearing mice; however after treatment, although the enzyme activity had a lower starting level, the pattern of depression of AP activity was similar (Figure 5a). Long term studies were not available as it was impossible to do these on the tumour-bearing mice.

*Serum lipids* Tumour-bearing mice developed marked lipaemia following CHIP treatment. However this was not detected in non-tumour-bearing or Neoplatin treated mice. Serum tryglycerides rose to eight times normal level appearing on Day 1 after CHIP treatment, reaching a maximum at Day 4, then decreasing slowly but still present after 15 days when the mice were sacrificed.

*Serum urea and creatinine* The effect of CHIP and Neoplatin on the serum urea and creatinine levels in normal and tumour-bearing male mice are shown in Figure 6. The serum urea levels showed a significant elevation after Neoplatin treatment in both normal and tumour-bearing mice. This rise was maintained and possibly increased up to the 60 days post treatment. There was no corresponding rise in the serum creatinine, in fact, the levels were

**Figure 5** Age relationship of serum alkaline phosphatase levels mean $\pm 1$ s.d. in female non-tumour-bearing mice treated with a single dose of Neoplatin (10 mg kg$^{-1}$(○)) or CHIP (40 mg kg$^{-1}$ (△)). Closed symbols show data for tumour-bearing animals. Serum was collected from 1 group of 10 animals sacrificed on each collection day (Day Number given in parenthesis) (a) over a Short Period post treatment (4–14 days) (b) over a Long Period post treatment (0–60 days). Control levels for untreated normal female mice were included for comparison (□).

slightly depressed at Day 7 post treatment (Day 9 in tumour-bearing mice) and then rose slightly.

In CHIP treated mice the serum urea levels only showed a slight increase around day 45 but then returned to within the normal range. On days 4 and 9 the serum creatinine was elevated. In the tumour-bearing mice on Day 1 post treatment there was an elevation in urea and creatinine above the normal range, but this soon returned to normal. A similar pattern was observed from the female mice.

*Urinary protein and N-acetyl-β-D-glucosaminidase (NAG) related to creatinine levels* Both urinary protein and urinary NAG levels were estimated and related to urinary creatinine levels in drug treated mice. The results for male mice are shown in Figure 7. After both drugs the levels of urinary protein were depressed well below the normal range and remained so throughout the period of investigation and this also applied to the level of urinary NAG in Neoplatin treated mice. In CHIP treated mice the NAG level appeared to remain normal for the first 20 days but then dropped sharply to a low level similar to that measured in the Neoplatin treated mice.

In the tumour-bearing mice the pattern is not so clear. There appears to be no depression below the control level established for the mice (Kind *et al.*, 1985) of urinary protein after either drug. After Neoplatin treatment, there was an initial rise in the level of urinary NAG and after CHIP treatment there was an initial fall but these levels soon returned to within the control ranges. A similar pattern was observed in female mice.

### Discussion

In any study of the toxic effects of chemotherapeutic agents *in vivo*, it is important to choose a drug dose which would be likely to elicit a response similar to that expected in a human patient. For example, if too high a drug dose is used, the toxic changes observed may only be those preliminary to

**Figure 6** Response of serum urea levels and serum creatinine levels in non-tumour-bearing and tumour-bearing male mice to treatment with a single dose of either Neoplatin (10 mg kg$^{-1}$ (○)) or CHIP (40 mg kg$^{-1}$ (△)). The control reference ranges (---) are taken from 10–20 week old mice. All values are mean ±1 s.d.

the death or severe incapacitation of the animal and are therefore not relevant to the clinical situation. The maximum tolerated dose (MTD) is a dose level which is routinely accepted as valid in the comparison and evaluation of different drugs *in vivo*. The MTD is defined as the largest dose where no deaths occur and the animal looks quite normal and healthy (i.e. no large weight loss etc).

The MTD of 40 mg kg$^{-1}$ CHIP was estimated from lethality studies and confirmed further by the use of the microcolony technique (Withers & Elkind, 1970). This is an assay which measures the percentage of crypts of lieberkuhn in the small intestine which are able to regenerate after increasing single doses of CHIP. This technique was developed to provide an assay of the cytotoxic effect of ionizing radiation on intestinal epithelial stem cells. Mice irradiated with large doses die of a gastro-intestinal syndrome which can be directly correlated with this cytotoxic effect on intestinal stem cells. The $LD_{50}$ dose of radiation is ~13 Gy in mice and this is a dose level at which only 50%

**Figure 7** Levels of urinary protein and urinary NAG related to creatinine after treatment of male mice with a single dose of either Neoplatin (10 mg kg$^{-1}$ (○)) or CHIP (40 mg kg$^{-1}$ (△)). The control reference ranges (---) are for 10–20 week old mice. All values are mean $\pm$1 s.d.

of the crypts of lieberkuhn are able to regenerate (Hamlet *et al.*, 1976). This technique is particularly relevant to the assessment of platinum drug toxicity where the mice also die of gastrointestinal toxicity. Therefore, the intestinal epithelium is a prime target for this drug. As can be seen in Figure 1, the MTD of 40 mg kg$^{-1}$ CHIP allows 99% of crypts to regenerate, whereas the $LD_{50}$ dose of 65 mg kg$^{-1}$ permits only 50% of the crypts to regenerate.

The gastrointestinal toxicity had previously been studied by the measurement of gastric distension after drug treatment (Jones & Stone, 1983). This distension is believed to indicate gastric toxicity in animals which do not show symptoms of nausea and vomiting. It can be seen from Figure 2 that even at the MTD CHIP induces a marked gastric distension commencing at ~7 h after drug treatment, reaching a maximum at 24 h and gradually returning to normal at about 4 days post treatment. The stomach volume remained within normal limits during the remainder of the fourteen days after treatment as did the weight of the animals. The biochemical changes from four days onward are therefore likely to be due to other causes.

### *Biodistribution of CHIP*

The biodistribution of CHIP has been studied by Hoeschele *et al.* (1984) using $^{195m}Pt$ labelled drug administered to normal female rats. They found the same sort of biphasic pattern as was found for the mice in Figure 3, with an initially rapid decline in radioactivity and then a much slower decline. Thatcher *et al.* (1982) compared the clearance of $^{191}$Pt labelled Neoplatin and CHIP from a small series of patients with malignant disease and also found a biphasic pattern of blood clearance which was the same for both drugs. On the other hand, the urinary excretion was greater for CHIP than for Neoplatin. They concluded that this difference might indicate that CHIP is potentially less nephrotoxic than Neoplatin.

Hoeschele *et al.* (1984) had compared the radioactivity in a number of rat tissues and found a much higher level in kidney than in other normal tissues, but in their rats the level in liver was intermediate unlike the high level shown in the mice in Figure 3. They noted that the level of CHIP in liver was twice as high as that of Neoplatin at 24 h after injection. Hepatotoxicity as well as nephrotoxicity might therefore have been expected after CHIP treatment. This was why the present study includes biochemical assays of both kidney and liver damage over a 60-day period, this allowing time for the damage after a MTD to become manifest in these organs where the cell populations have much slower kinetics than in the intestine.

The initially high level in the small intestine (Figure 3) would account for the intestinal toxicity manifest in the crypts of lieberkuhn (Figure 1) and the early onset of lethality when higher drug dosage was used.

Hoeschele *et al.* (1964) studied normal rats whereas the data in Figure 3 apply to tumour-bearing mice. The uptake of CHIP into the tumour eventually rose to the same level as found in the small intestine and the blood. This confirms the potential of CHIP for cancer chemotherapy and also as a radiosensitizing agent (Laverick & Nias, 1981).

From the initial toxicity data and radioactive incorporation of platinum, it can be seen that the liver and kidney are organs which may be prime targets for the two platinum drugs when used at a non-lethal dose level. Therefore, renal function and liver function were analysed in greater detail using the biochemical tests described after the treatment of the mice with a single MTD of either drug.

### *Hepatotoxicity*

There are no reports of liver damage from platinum drug administration but equally high concentrations of CHIP were found in the liver as in the kidney. Hence appropriate enzyme assays were performed to assess liver involvement.

*Serum transaminase activity* The marked differences seen in levels of serum transaminase for non-tumour bearing and tumour-bearing mice have already been reported (Kind *et al.*, 1985). The higher AsT level found in the tumour bearing mice (when compared with non-tumour bearing mice of the same age) could be consistent with some degree of hepatotoxicity as in humans. This could be the result of damage to hepatocytes, the enzymes leaking out from the cell either due to changed permeability or due to cell necrosis. It could be postulated that the absence of any change in transaminase levels in the drug treated tumour and non-tumour-bearing mice may not therefore necessarily exclude some toxic effect of platinum as suppression of protein synthesis is known to occur with this drug, thus masking any rise due to toxicity. The platinum complexes bind avidly to proteins, and they are known to slow down the rate at which cells pass through the cell cycle and cause delay of cells entering into mitosis or at the Gl/S boundary of the cell cycle (Szumiel & Nias, 1976; Barot *et al.*, 1985). This could well be a direct or indirect effect of altered intracellular enzyme levels.

*Alkaline phosphatase* In humans a rise in AP levels may indicate hepatobiliary dysfunction. However, phosphatase of bone origin must also be taken into consideration when assessing results. In normal mice, it has been shown that AP levels are age related (Figure 4). After treatment with CHIP or Neoplatin the AP levels were depressed in non-tumour-bearing mice and remained so throughout the period of study, (Figure 5). Although tumour-bearing mice initially had lower levels of AP than non-tumour-bearing mice, the pattern of change after CHIP and Neoplatin was similar to that for untreated mice (Figure 5a).

Isoenzyme studies (Kind unpublished data) suggest that the alkaline phosphatase was mainly of bone origin. Whole body autoradiography of $^{195m}$Pt labelled Neoplatin in non-tumour-bearing and tumour-bearing mice shows a high persisting concentration of platinum in cartilage and bone (Benard *et al.*, 1983). Such a deposition in the non-tumour-bearing mice may induce damage to the osteoblasts with a resultant reduction in alkaline phosphatase activity. In the tumour-bearing mice, there is a possibility that the bone enzyme may already be markedly depressed and the enzyme activity seen after drug treatment may be mainly that of liver origin which would remain unchanged provided there was no cholestatic liver involvement. The depression of AP was noted to be more marked after Neoplatin in the non-tumour-bearing animals.

*Serum lipid changes*

The marked lipaemia observed after CHIP treatment of tumour-bearing mice was not seen in non-tumour-bearing mice. This may indicate that the enzyme, lipoprotein lipase, which is responsible for the catabolism of circulating triglyceride-rich lipoproteins (chylomicrons and very low density lipoprotein), may be suppressed in tumour-bearing mice, and that this effect is enhanced by CHIP treatment.

*Nephrotoxicity*

Nephrotoxicity after Neoplatin therapy is well recognised in clinical practice and experimental animals, producing both glomerular and tubular damage. In their clinical study Campbell *et al.* (1983) measured creatinine clearance but in many rodent studies, the assay of nephrotoxicity is confined to estimates of blood urea nitrogen (Levi *et al.*, 1982; Uozomi *et al.*, 1983; Osman *et al.*, 1984) although Shepherd *et al.* (1980) also assayed urinary protein as well. In this study the biochemical tests used to monitor glomerular function included both the measurement of serum urea and creatinine, urinary protein to indicate any proteinuria present, and urinary NAG activity as a sensitive indicator of tubular damage.

*Serum urea and creatinine* The persistently raised serum urea levels after Neoplatin treatment confirmed the nephrotoxic effect of this drug (Figure 6). By contrast, non-tumour-bearing mice treated with CHIP showed only a transient rise in serum urea at about Day 45 after treatment. Harrison *et al.* (1983) have shown that CHIP has a greater retention time in the tissues than Neoplatin and there is a secondary uptake by the kidney, presumably as a result of the delayed release from the tissues. The increased serum urea levels on Day 45 in CHIP treated mice may reflect a greater retention and a subsequent late release of CHIP from the tissues. It must be pointed out that the mice in this study received a single dose of CHIP or Neoplatin albeit at the MTD. The possibility, therefore, of long term toxicity after repeated drug administration cannot be ruled out.

In humans a rise in serum urea is usually accompained by a parallel rise in serum creatinine. However, no rise in serum creatinine was observed. It was not practicable to carry out 24 h creatinine clearance studies on the mice as is usually carried out in human patients for assessment of renal function. This may have given a better measurement of glomerular function. Usually as many as 50% of the functional nephrons will have to be lost before serum creatinine levels begin to rise appreciably. The lack of creatinine elevation may indicate that the raised urea levels may be the result of an extra renal mechanism such as increased tissue catabolism or dehydration. However, we observed neither persistent weight loss nor signs of dehydration in the treated mice. Furthermore, in mice, the urine creatinine, creatine ratio is 1:1 and the metabolism of creatinine and creatine is probably different from that in humans where the ratio is 100:1. Therefore, in mice, a rise in serum creatinine may be a less sensitive indicator of impaired glomerular function.

*Urinary protein and NAG* The C3H mouse normally excretes considerable quantities of protein in the urine. This protein is though to be a 'sex' protein with the electrophoretic mobility of prealbumin and a molecular weight of $\sim$18,000 daltons (Finlayson & Baumann, 1958). Most of this protein is filtered through the glomerulus and it would be expected that some of the protein would be reabsorbed. The presence of this sex protein means that changes in the excretion of other proteins associated with glomerular and tubular damage may be masked, only to be identified by more specific immunological techniques.

No increase in urinary protein excretion was observed in any of the animal groups to indicate glomerular or tubular dysfunction. In untreated tumour-bearing mice, total urinary proteins are decreased (Kind *et al.*, 1985) and these levels remained unaltered after treatment with CHIP or Neoplatin. However when non-tumour-bearing mice are treated with CHIP or Neoplatin, there was a marked decrease in protein content, falling to levels similar to those observed in the untreated tumour-bearing mice (Figure 7). This decrease in excretion could again be an indication of a decrease in protein synthesis.

The reason why the tumour-bearing mice did not show further reduction in urinary protein levels after drug treatment is unclear, but may indicate that the synthesis of the sex protein is the one mainly affected by the drug treatment and cannot be suppressed further in mice with already depressed protein levels. Kind *et al.* (1985) found that in fact the total serum proteins were significantly lower in untreated tumour-bearing mice and it could be postulated that the sex protein might also have been affected as well as these animals.

The fall in urine NAG activity in male mice treated with CHIP was less apparent than in those treated with Neoplatin, the level in the mice of the former group not falling until Day 20. The patterns were similar but less marked in the females. However, the tumour-bearing mice showed initially increased NAG activity after Neoplatin, indicating some possible cell damage, (Figure 7). The increase may have been even greater in the male tumour-bearing mice as they had some depression of NAG levels prior to treatment.

The pattern of change for NAG was similar in most cases to that for urine protein, and this may be important in the interpretation of results. NAG activity has been shown to correlate well with protein levels in normal mouse urine, both being much higher in males (Kind *et al.*, 1985). The source of urinary NAG is the lysosomes of the proximal tubular cells, and that of protein most likely from the plasma by glomerular filtration. It is postulated that the load of protein in the proximal tubule may elicit a lysosomal response for its reabsorption, hence NAG being released. If urine protein falls then NAG activity may be reduced. Therefore the fall in activity seen after CHIP and Neoplatin in normal mice may be related to both a decreased protein load and decreased synthesis of NAG in the kidney. Thus any release of enzyme due to toxicity may be masked.

It was interesting to observe that in the tumour-bearing mice there was a rise in NAG activity after Neoplatin, however there was no change in protein which might otherwise influence NAG levels? Could the presence of the tumour make the renal tubular cells more susceptible to the toxic effects of Neoplatin?

In conclusion, this study has shown that the pre-clinical evaluation of the biochemical effects of cancer chemotherapeutic agents should be undertaken in tumour-bearing as well as normal animals. Furthermore, the MTD of the drug should be used so as to allow a reasonably long period of observation after treatment. Unexpected renal and hepatic changes have been revealed which draw attention to additional mechanisms of drug toxicity that may occur in clinical practice. The study confirms that the second generation platinum coordination complex CHIP is less toxic than the original complex Neoplatin.

We are grateful to Mr M.G. Stone for his technical assistance. M.L. and M.G. were supported by grants from the Cancer Research Campaign and May & Baker Ltd, respectively. We thank Mrs Nikki Tucker for processing the manuscript.

### References

BAROT, H.A., LAVERICK, M. & NIAS, A.H.W. (1985). The radiomimetic properties of a platinum drug. *Br. J. Radiol.*, **58**, 51.

BENARD, P., DESPLANCHES, G., MACQUET, J.P. & SUMON, J. (1983). Whole-body autoradiographic study of the distribution of $^{195m}$Pt in healthy and tumor-bearing mice treated with labelled cisplatin. *Cancer Treatment Rep.* **67**, 457.

CAMPBELL, A.B., KALMAN, S.M. & JACOBS, C. (1983). Plasma platinum levels: Relationship to cisplatin dose and nephrotoxicity. *Cancer Treatment Rep.* **67**, 169.

CREAVEN, P.J. MADAJEWICZ, S., PENDYALA, L. & 5 others. (1983). Phase I Clinical trial of cis-dichloro-transdihydroxy-bis-isopropylamine platinum (IV) CHIP. *Cancer Treatment Rep.* **67**, 795.

DENTINO, M., LUFT, F.C., YUM, M.N., WILLIAMS, S.D. & EINHORN, L.H. (1976). Long term effect of cis-diammine-dichloro-platinum (CDDP) on renal function and structure in man. *Cancer*, **41**, 274.

FINLAYSON, J.S. & BAUMANN, C.A. (1958). Mouse proteinuria. *Am. J. Physiol.*, **192**, 69.

HAMLET, R., CARR, K.E., TONER, P.G. & NIAS, A.H.W. (1976). Scanning electron microscopy of mouse intestinal mucosa after cobalt 60 and D-T neutron irradiation. *Br. J. Radiol.*, **49**, 624.

HARRISON, R., McAULIFFE, C.A., ZAKI, A. & 5 others. (1983). A comparative study of the distribution in the male rat of platinum-labelled cis-dichlorodiammine Platinum (II), cis-trans-dichlorodihydroxy-bis-(Isopropylamine) Platinum (IV), and cis-dichoro-bis-cyclopropylamine Platinum (II). *Cancer Chemother. Pharmacol.*, **20**, 90.

HOESCHELE, J.D., FERREN, L.A., ROBERTS, J.A. & WHITFIELD, L.R. (1984). Biodistribution and pharmacokinetics of $^{195m}$Pt-labelled CHIP in the normal female Fischer 344 rat in platinum coordination complexes. In *Cancer Chemotherapy*, Hacker, M.P., Douple, E.B. & Krakoff, L.H. (eds) p. 103. Martinus Nijhoff Publishing: Boston.

JONES, B. & STONE, M.G. (1983). Mechanisms of gastric distension following cytotoxic drugs. Effects of metoclopramide. *Br. J. Cancer*, **48, 128.**

KIND, P.R.N., GORDON, M., LAVERICK, M., NIAS, A.H.W. & SLAVIN, B.M. (1985). The effect of C3H mouse mammary tumour on levels of serum and urine analytes *in vivo*. *Br. J. Cancer*, **52**, 607.

LAVERICK, M. & NIAS, A.H.W. (1981). Potentiation of the radiation response of hypoxic mammalian cells by CHIP. *Br. J. Radiol.*, **54**, 529.

LEVI, F., HRUSHESKY, W.J.M., BORCH, R.F., PLEASANTS, M.E., KENNEDY, B.J. & HALBERG, F. (1982). Cisplatin urinary pharmacokinetics and nephrotoxicity: A common circadian mechanism. *Cancer Treatment Rep.*, **66**, 1933.

OSMAN, N.M., COPLEY, M.P. & LITTERST, C.L. (1984). Amelioration of cisplatin-induced nephrotoxicity by the diuretic acetazolamide in F 344 rats. *Cancer Treatment Rep.*, **68**, 999.

SHARMA, H.L. & SMITH, A.G. (1981). The short-lived radioisotope production program at Manchester. *J. Radioanalyt. Chem.*, **64**, 249.

SHEPHERD, R., KUSNIERCZYK, H., JONES, M. & HARRAP, K.R. (1980). Criteria for the selection of second generation platinum compounds. *Br. J. Cancer*, **42**, 668.

SZUMIEL, I. & NIAS, A.H.W. (1976). Action of a platinum complex cis-dichloro-bis cyclopentylamine Pt II on Chinese hamster ovary cells *in vitro*. *Chem. Biol. Interact.*, **14,** 217.

THATCHER, N., SHARMA, H., HARRISON, R. & 5 others. (1982). Clearances of three radioactively labelled platinum complexes. *Cancer Chemother. Pharmacol.*, **9,** 13.

TOZER, G.M., PENHALIGON, M. & NIAS, A.H.W. (1984). The use of ketamine and diazepam anaesthesia to increase the radiosensitivity of a C3H mouse mammary adenocarcinoma in hyperbaric oxygen. *Br. J. Radiol.*, **57,** 75.

UOZOMI, J., SAGIYAMA, K., AOKI, K., IWAMATO, Y. & BABA, T. (1983). Effectiveness of 'two route chemotherapy' using cisplatin and its antidote, sodium thiosulfate, on lifespan of rats bearing metastatic liver tumors. *Cancer Treatment Rep.*, **67,** 1067.

WILTSHAW, E. (1978). A review of clinical experience with cis-platinum diammine dichloride. *Biochimie,* **60,** 925.

WITHERS, H.R. & ELKIND, M.M. (1970). Microcolony survival assay for cells of mouse intestinal mucosa exposed to radiations. *Int. J. Radiat. Biol.*, **17,** 261.